# Research on the Influence Mechanism of Consumers’ Perceived Risk on the Advertising Avoidance Behavior of Online Targeted Advertising

**DOI:** 10.3389/fpsyg.2022.878629

**Published:** 2022-04-05

**Authors:** Hai Jian Wang, Xia Lei Yue, Aisha Rehman Ansari, Gui Qian Tang, Jian Yi Ding, Ya Qiong Jiang

**Affiliations:** ^1^School of Business, Guilin University of Electronic Technology, Guilin, China; ^2^Department of Management Sciences, GIFT University, Gujranwala, Pakistan

**Keywords:** online targeted advertising, advertising avoidance, perceived risk, negative emotions, COVID-19 pandemic

## Abstract

In China, online sales continue to grow against the generally adverse effects of the COVID-19 pandemic on economic development. Although advertisers favor online targeted advertising for its precision, consumers may find it intrusive and avoid it. This study constructed a conceptual model based on Stimulus-Organism-Response (SOR) theory, Approach-Avoidance Theory, and Brand Avoidance Theory to investigate the influence mechanism of consumers’ perceived risk on the avoidance behavior of online targeted advertising via an online survey. Collected 436 validated data was analyzed through structural equation method in AMOS statistical software. Results showed that the positively influenced advertising avoidance, and negative emotions mediated the relationship between perceived performance risk, time-loss risk, freedom risk, and advertising avoidance, but perceived privacy risk did not influence advertising avoidance through negative emotions. Perceived COVID-19 risk moderates the effect of negative emotions on advertising avoidance. The findings provide important insights for helping governments, advertisers and online platforms into which risk perceptions influence advertising avoidance, and suggests ways to mitigate consumers risk perceptions for the mutual benefit of brands and users.

## Introduction

Internet enabled e-commerce enables businesses to expand, and has significantly changed marketing environments (Masoud, 2013; Nair, 2017). Enterprise digital transformation has promoted the digitalization of marketing methods (Li et al., 2020). The scale of online advertising exceeds that of traditional broadcast and printed media (Wijenayake and Pathirana, 2019). Global digital advertising spending reached $332.84 billion in 2020, up 2.4% from 2019 (eMarketer, 2020), online targeted advertising accounts for 59% of the market (Lin, 2021). In China in 2020, advertising revenue from printed media, broadcasting and online was 872.9 billion Yuan, an increase of 12.9% over 2019; coming mainly from the expansion of online advertising. In 2020, the online advertising market reached 766.6 billion Yuan, up 18.6% year-on-year; online advertising accounted for 87.2% of all advertising revenue; and 76.6% of advertisers increased their online advertising spending (iResearch, 2021). Online advertising employs user datasets to infer user demographics, habits and preferences, thereby enabling advertising to be targeted more precisely (Wijenayake and Pathirana, 2019; Aiolfi et al., 2021; Li and Yin, 2021). Targeting consumers is also regarded as the essential difference between online and traditional advertising (Goldfarb, 2014). Targeted advertising refers to “Relying on the Internet advertising network and advertising trading platform, we apply big data information retrieval, audience targeting and data mining technologies to capture and analyze target consumers data in real time, and push out highly relevant commercial information in response to consumers’ personalized characteristics and needs (Ju et al., 2015).” Targeted advertising delivers suitable advertising messages to consumers at the right time in order to convert users’ potential needs into actual needs (Adam, 2002; Philip and Gary, 2007). Compared to traditional advertising, targeted advertising shows more than twice the click-to-buy conversion rate (Fullerton et al., 2019).

Advertisers choose targeted advertising for marketing refinement and efficiency. Online targeted advertising is favored by advertisers due to its high matching degree with consumers’ needs. The “home economy” and “zero-contact services” associated with COVID-19 have created higher online views and larger consumer groups for online advertising (Yao and Shang, 2021). Numerous studies show that targeted online advertising can help increase brand awareness (Drèze and Hussherr, 2003; Niu et al., 2021), brand recognition (Keller, 2010), and influence purchasing intentions (Lewis and Reiley, 2014). However, not all consumers respond positively to online advertising, are increasingly aware that it is aggressive and causes negative cognition and emotions (Wijenayake and Pathirana, 2019; Youn and Kim, 2019), leading many consumers to try to block advertising (Verlegh et al., 2015; Dodoo and Wen, 2019). An advertising industry report showed that 82% of Americans avoid online advertising (OneSpot, 2021). Advertising avoidance may be defined as “all actions by media users that differentially reduce their exposure to advertising content” (Speck and Elliott, 1997). Advertising avoidance works against attempts to reduce marketing costs through online targeted advertising by reducing the return on advertising costs (Ma and Pan, 2020) and threatening the survival and development of enterprises. Therefore, advertisers proposed marketing practices to reduce advertising avoidance by engaging consumers (Akram et al., 2017b; MarTech Series, 2021), such as creating headlines that draw their attention (Swatmarketing Solutions, 2021). Although these practices are helpful in some situations, they cannot address the fundamental reasons why consumers avoid online advertising. Therefore, it is important to explore how to make good use of the positive effects of online targeted advertising while minimizing advertising avoidance, and to understand what factors trigger avoidance behavior when consumers are exposed to online targeted advertising. In order to improve the effectiveness of online targeted advertising and improve users’ advertising experience, we propose adjustment and improvement measures for these influencing factors, so as to improve the return on investment of advertising and promote mutual benefits for brands and users.

In line with approach-avoidance theory (Elliot, 2006), people avoid advertising because they perceived negative outcomes (Kelly et al., 2018). Perceived risk, as the core motivator in consumer behavior, is considered the key obstacle to the development of e-commerce (Malaquias and Hwang, 2016). Understanding and reducing risk perceptions of consumers who simply browse products or services on the Internet is critical when transforming them into effective buyers (Speck and Elliott, 1997). Despite more attention being paid to online targeted advertising avoidance, there is a scarcity of research on online targeted advertising avoidance. Researchers mainly combine Transaction Cost Theory (TCT), Theory of Rational Behavior (TRA), and Technology Acceptance Model (TAM) to explore the mechanism of advertising avoidance. It is found that targeted advertising has a negative effect on advertising avoidance from perceived usefulness (Zanot, 1984; Shin and Lin, 2016), perceived entertainment (Edwards et al., 2002; Kelly et al., 2013; Shin and Lin, 2016), advertising authenticity (Li and Ye, 2006), platform credibility (Li and Ye, 2006), perceived convenience (Okazaki et al., 2012), advertising interactivity (Kelly et al., 2013) and advertising relevance (Kelly et al., 2013). Perceived goal impediment (Cho and Cheon, 2004; Li and Ye, 2006; Shin and Lin, 2016), advertising clutter (Cho and Cheon, 2004), past negative experience (Cho and Cheon, 2004; Shin and Lin, 2016), privacy concerns (Barnes, 2006), perceived cost (Shin and Lin, 2016) and other factors positively affect advertising avoidance. Mitchell argues that consumers tend to minimize risk rather than maximize utility when making decisions (Mathieson, 1991). Therefore, measures to reduce online targeted advertising avoidance can be found only by understanding what risks consumers perceive and avoid online targeted advertising. Literature on advertising avoidance from the perspective of risk perception mainly focuses on perceived privacy risks (Barnes, 2006; Chen et al., 2019; Labrecque et al., 2021), but in addition to perceived privacy risk, there is also advertising inauthenticity (Ducoffe, 1996) or lack of integrity (Kelly et al., 2013) will cause the performance risk, the time-loss risk caused by homogenous advertising push and the freedom risk from obstacles to finding targets (Li and Lee, 2002), and health risks due to the outbreak of the COVID-19. However, few studies integrate the various perceived risks into a single framework to explore the influencing factors that lead to user online targeted advertising avoidance from the perspective of consumers.

According to brand avoidance theory (Knittel et al., 2016), negative emotions generated by consumers can reduce positive attitudes toward advertised products (Steenkamp and Hans, 1992). And risk perception can cause negative emotions (Finucane et al., 2000; Yan et al., 2015), which can lead to advertising avoidance (Li, 2021). During the COVID-19 pandemic, online targeted advertising has become one of the main sources of information for consumers. Therefore, it is worth exploring whether negative emotions mediate perceived risk and advertising avoidance, and whether perceived COVID-19 risk moderates the effect of negative emotions on advertising avoidance.

The study adopted a questionnaire method to conduct empirical analysis, combined perceived risk theory and SOR theory, and innovatively introduces perceived risk, negative emotions, and adverting avoidance into the research on the impact mechanism of online targeted adverting avoidance. Specifically, this study is aim to explore the relationship between perceived risk and adverting avoidance, and verify the mediating role of negative emotions, and moderating role of perceived COVID-19 risk between negative emotions and adverting avoidance. The study helps to expand the vision of online targeted adverting avoidance, better explain the impact mechanism of online targeted adverting avoidance, reduce consumer avoidance behaviors and improve the effectiveness of online targeted adverting, provide important strategic guidelines on how to better promote healthy development of online targeted adverting business models, and bring more value to society and consumers.

The rest of the article is structured as follows: Part II defines relevant concepts and describes the current state of research. Part III describes theoretical underpinnings and presents the study hypotheses and model. Parts IV and V describe instrument development, data sources and mathematical and statistical results of the questionnaire data and data analysis. Part VI analyzes the results and discusses theoretical and practical implications and limitations of the study.

## Literature Review

### Adverting Avoidance

Advertising avoidance is defined as “all actions that media users employ to reduce exposure to advertising content” (Speck and Elliott, 1997). Advertising avoidance is a subordinate study to advertising effectiveness research, and most studies draw on psychological research paradigms and methods. Advertising avoidance can be classified as behavioral, cognitive, or affective (Speck and Elliott, 1997; Cho and Cheon, 2004; Kelly et al., 2018). The delimitation of this study advertising avoidance is behavioral avoidance. This study defined advertising avoidance as the behavior adopted to avoid exposure to advertisements, by interrupting the communication of an advertisement in this study (Cho and Cheon, 2004), such as by scrolling down a web page to close the advertisement, and other actions.

Research into advertising avoidance extends into its complexities, including advertising avoidance under time pressure (Duan and Wu, 2021); perceived goal impediments (Cho and Cheon, 2004; Li and Ye, 2006; Shin and Lin, 2016), perceived advertising clutter (Cho and Cheon, 2004), previous negative experiences (Cho and Cheon, 2004; Shin and Lin, 2016), perceived usefulness (Zanot, 1984; Shin and Lin, 2016), and perceived relevance (Kelly et al., 2013). Research has gone through the three stages of traditional media, web 2.0 and web 3.0, and its influencing factors have also undergone significant transformation. With the realization of user orientation in the web 3.0 era, under the influence of the technological wave of artificial intelligence and machine learning and humanistic thinking, advertising avoidance research has effectively returned to a focus on people, and more and more researchers are studying users’ behaviors and attitudes toward advertising from the perspective of their perceived benefits and risks (Chen and Duan, 2021). Knittel et al. (2016) suggests that scholars can identify which advertising-specific factors influence advertising avoidance. This could help marketing managers plan their advertising campaigns and reduce the risks associated with expensive advertising. Therefore, this study investigated the mechanism of consumers’ risk communication on advertising avoidance from the perspective of their perceptions, in order to propose insights for weakening the risk perceptions brought by targeted advertising, in order to narrow the research gap and resolve consumers’ ambivalence of both wanting to enjoy convenience and fearing to bear losses, so as to reduce consumers’ advertising avoidance.

### Perceived Risk

Bauer (1960) introduced the concept of “perceived risk” from psychology into consumer research. Mangold and Faulds (2009) asserted that perceived risk is defined as the uncertainty of services or goods. The present study defines perceived risk as an uncertainty regarding the possible negative consequences of using Online Targeted Advertising (Zhang, 2019).

Studies show that perceived risk is a multidimensional construct (Kaplan et al., 1974; Stone and Grønhaug, 1993; Forsythe and Bo, 2004; Chen, 2020). Cunningham (1967) classified consumer perceived risks as financial, psychological, performance, physical, time-loss, and social; a six-dimensional model of consumer risk perception. In the study of perceived risk, scholars should reconstruct their research models based on the six-dimensional model of perceived risk according to the research context (Campbell and Goodstein, 2001). In the online environment, studies have found physical (Jacoby and Kaplan, 1972), social (Barnes, 2006; Yu et al., 2007; Faqih and Jaradat, 2015), and psychological risk (Forsythe and Bo, 2004; Shao et al., 2006) to be redundant. However, the online environment can threaten consumer privacy, making privacy risk an important dimension of perceived risk (Miyazaki and Fernandez, 2001; Chen et al., 2019; Labrecque et al., 2021). Therefore this study omitted physical, psychological and social risks and added the perceived privacy risk dimension. Combined with the online targeted advertising research context, this study is concerned with advertising avoidance rather than purchase behavior and intention, so this study combines financial and performance risks into one dimension of performance risk, as suggested by Chen (2020). More importantly, studies have found that targeted advertising influences consumers’ decision quality and effort (Xiao and Benbasat, 2018), with limited capacities to choose and buy (Aguirre et al., 2015; Newell and Marabelli, 2015), the opportunity to choose (Chen et al., 2019), and the perception that choices are manipulated (Fan et al., 2020). In particular, when consumers use the search function, the platform prioritizes advertisements from merchants that have purchased priority, and consumers’ ability to freely access information is limited by the pushing of precise advertisements (Newell and Marabelli, 2015; Chen et al., 2019). Consumers feel limited in their search results. Such restrictions can be seen as a threat to freedom in the Internet age (Liu, 2019). According to resistance theory (Chao et al., 2020) “When freedom is threatened or restricted, people are generally motivated to restore it.” This study proposes that perceived freedom risk is a new dimension of perceived risk. Therefore, combining the literature on dimension of perceived risk, and considering the characteristics of online targeted advertising, this study selected the four dimensions of perceived performance risk, perceived privacy risk, perceived time-loss risk, and perceived freedom risk to investigate the influence of perceived risk on advertising avoidance. The definition of each dimension is shown in Table 1.

**TABLE 1:** Definitions of perceived risks dimensions.

Dimensions	Definitions	Source
Perceived performance risk	The “possibility of the product malfunctioning and not performing as it was designed and advertised and therefore failing to deliver the desired benefits” and “concern over any financial loss that might be incurred because of online Targeted advertising”	Grewal et al., 1994; Guru et al., 2020
Perceived privacy risk	The risk of losing control over personal information when user information is used without their permission	Park et al., 2004
Perceived time-loss risk	The “amount of time required to browse and compare advertising messages and time and effort lost in returning or exchanging the product”	Nai-Yi and Zhou, 2014; Guru et al., 2020
Perceived freedom risk	The limitation of consumer freedom to search for information or to choose	Liu, 2019; Youn and Kim, 2019

In recent years perceived risk has been widely studied in consumer behavior and attitudes. The literature has focused on online purchasing intentions (Pelaez et al., 2019; Ahmed et al., 2021; Jain, 2021), buying behavior (Wai et al., 2019; Tobi et al., 2020) impulse buying (Wu et al., 2020) travel behavior (Matiza and Kruger, 2021; Sharma et al., 2021), and customer loyalty (Esmaeili et al., 2021). Perceived risk has been less studied in the advertising industry. Perceived costs, privacy concerns, and the opportunity costs of online targeted advertising can cause consumers to resist online targeted advertising (Chen et al., 2019). Chao et al. (2020) studied the relationship between perceived privacy risk, psychological resistance and attitude to advertising, and found that perceived privacy risk had no significant effect on advertising attitude; Li et al. (2016) and Kurtz et al. (2021) studied location-based advertising (LBA) and found that perceived risk has an influence; in short video situational advertising, Zhang Meizhen confirmed the positive effect of perceived risk on psychological resistance and thus on consumers’ willingness to accept through the SOR model. The literature shows that most applications of perceived risk theory focus on the effects on online shopping intention or behavior (Pelaez et al., 2019; Wai et al., 2019; Tobi et al., 2020; Wu et al., 2020; Ahmed et al., 2021; Jain, 2021; Matiza and Kruger, 2021; Sharma et al., 2021). There is less research on advertising avoidance in online targeted advertising, and there are inconsistencies in conclusions (Chen et al., 2019; Chao et al., 2020). Thus there is need to identify the impact of perceived risk on advertising avoidance in online targeted advertising.

### Negative Emotions

The psychological concept of emotion refers to an individual’s reaction to a specific object in response to external stimuli. Negative emotions include anger, complaints, depression, regret, and helplessness (Jaeger et al., 2019). In this paper negative emotions are defined as negative feelings such as anxiety and anger that consumers have in response to targeted advertising (Huang, 1997). Behavior is considered as an emotion-centered coping mechanism (Akram et al., 2021). When consumers find services on a website abruptly, serendipitous information from discovering information can be elicited to their emotional affection (Umair et al., 2018). When processing information about products consumers use their emotions as a measure (Grau and Folse, 2007). Emotions play an important role in the prediction of advertising effectiveness (Edell and Burke, 1987) and can influence its effectiveness (Petty et al., 1988; Zhang et al., 2015). Negative emotions have been widely used as an important mediating variable in the study of consumer behavior and attitudes. Negative emotions mediate between cognition and consumer behaviors and attitudes (Liu and Wang, 2017; Li, 2021).

## Research Model and Hypothesis Development

### Underpinning Theory and Proposed Framework

#### Stimulus-Organism-Response Theory

**FIGURE 1:**
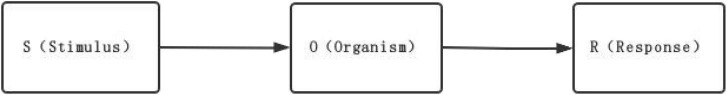
The model of the SOR theory.

Stimulus-Organism-Response theory refers to the behavioral responses that individuals produce through mental processing in response to stimuli from external environmental factors. Stimuli are the influencing factors that produce emotions; organism refers to mental processing after being stimulated, such as changes in emotions and attitudes; and response is the behavioral response after processing (Mehrabian and Russell, 1974). The SOR model (Figure 1) can clearly explain the influence mechanism of individual behavior.

Stimulus-Organism-Response theory was first applied by Belk (1975) in marketing research. After that, the theory was widely used in the study of consumer attitudes and behaviors because of its generalizability and extensibility (Akram et al., 2020). Many researchers use SOR theory or combine it with other theories when studying consumer advertising avoidance. For example, Wan et al. (2015) and Zhang (2019) combined psychological reactance theory and reverse psychology theory to study the psychological mechanisms that lead avoidance of advertising. Using the theory of planned behavior, Liu and Fu (2014) investigated perceived risk influences mobile advertising avoidance through the trust path. Using this theory Ryu and Park (2020) showed that perceived advertising persuasiveness affects advertising avoidance. The rational choice of elements of S, O, and R is beneficial to improve descriptions of reality. Lai (2010) used perceived risk as a stimulus to study consumer behavior Gao et al. (2017) studied consumer avoidance or convergence mechanisms by emphasizing the role of emotion as a mediator of the organism in external environmental stimuli and customer purchase behavior. Kim et al. (2007) found that adding moderating variables to the SOR model enhanced the strength of the model to explain reality. Song et al. (2017) verified the moderating role played by consumer product type in explaining different tendencies of consumers to shield themselves from different personalized advertisements. Jiang (2017) added privacy concerns as a moderating variable in order to explain the contradictory relationship between consumers’ perceived usefulness and consumers’ attitudes.

This study adopts SOR theory as an underpinning theory to investigate the mechanism of consumers’ avoidance of targeted advertising. The dimensions of perceived performance, privacy, time-loss risk and freedom risks are selected as Stimulus, negative emotion as Organism, and consumers’ advertising avoidance as Response.

#### Approach-Avoidance Theory

Approach-Avoidance Theory (Roth and Cohen, 1986) argues that approach and avoidance are mental and emotional activities that are oriented toward or away from the threat. Approach motivation refers to the choice to move closer toward the behavior or direction of a positive stimulus (objects, events, and possibilities), whereas avoidance motivation is the choice to move away or flee toward the behavior or direction of the behavior of a negative stimulus (objects, events, and possibilities) (Elliot, 2006). In this study, perceived risk is considered a negative stimulus under which consumers may choose to stay away or to avoid behavior (advertising avoidance).

#### Brand Avoidance Theory

Advertising avoidance is an important dimension of brand avoidance. Brand avoidance theory suggests that consumers develop advertising avoidance when they have negative emotions about advertisements, such as annoyance or irritation, but it does not provide much of an explanation for why consumers develop negative emotions (Knittel et al., 2016).

#### Research Framework

**FIGURE 2:**
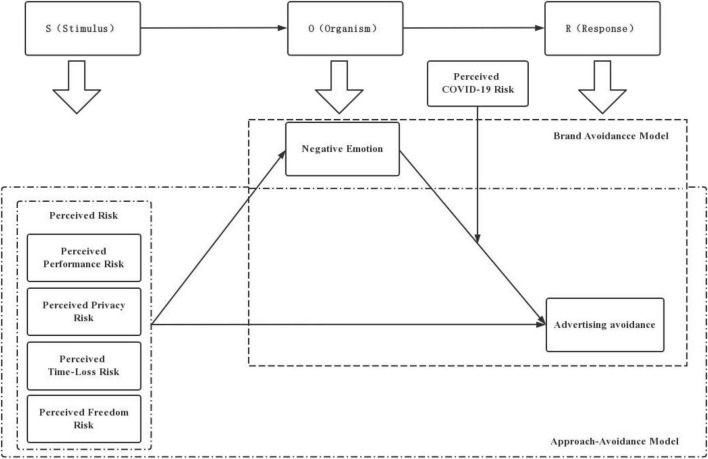
Theoretical framework.

To study the influence of perceived risk on advertising avoidance this study used a model that combined SOR theory, Approach-avoidance Theory, and brand avoidance theory, and the research framework of the. The model is shown in Figure 2.

Perceived performance risk, perceived privacy risk, perceived time-loss risk and perceived freedom risk were selected as the four dimensions of perceived risk as independent variables; adverting avoidance as dependent variable; negative emotions as mediator, and perceived COVID-19 risk as moderator. The study investigated the relationships between the four constructs of perceived risk and adverting avoidance; the mediating role of negative emotions in the relationship between the four constructs of perceived risk and adverting avoidance, and the moderating role of perceived COVID-19 risk in the relationship between negative emotions and adverting avoidance.

### Hypotheses Development

#### Perceived Risk and Advertising Avoidance

According to Approach-avoidance Theory, when users perceive risk in online targeted advertising, they develop avoidance motivation and choose to avoid the advertisement (Elliot, 2006). When consumers subjectively perceive that a product or service does not meet the minimum requirements, it causes them to perceive risk and adopt avoidance behavior to reduce their exposure to that risk (Taylor, 1974). Researchers have found that online targeted advertising overstates product effectiveness, provides ineffective or false information, over-collects and uses users’ private information, wastes users’ time, limits their freedom of search and choice, and generates negative behaviors and attitudes when users perceive that these risks do not match their requirements (Mitchell, 1999; Newell and Marabelli, 2015; Chen et al., 2019; Pelaez et al., 2019; Wai et al., 2019; Tobi et al., 2020; Wu et al., 2020; Ahmed et al., 2021; Jain, 2021; Matiza and Kruger, 2021; Sharma et al., 2021). Therefore, the study proposes the following hypothesis:

H1: Perceived risk has a positive effect on advertising avoidance.

H1a: Perceived performance risk has a positive effect on advertising avoidance.

H1b: Perceived privacy risks has a positive effect on advertising avoidance.

H1c: Perceived time-loss risks has a positive effect on advertising avoidance.

H1d: Perceived freedom risks has a positive effect on advertising avoidance.

#### The Mediating Role of Negative Emotions

Online targeted advertising is convenient for consumers because of its precise targeting of users’ needs, but being “too precise” may cause uneasiness and produce negative emotions (Chen et al., 2019). Inappropriate and exaggerated information and excessive advertising will enhance user stress and lead to negative emotions such as disgust (Che et al., 2019; Chen et al., 2021). Consumers perceive the risk of targeted advertising and uncertainty about the outcome will trigger negative emotions, making individuals doubtful, fearful and angry (Li et al., 2011, 2016; Smit et al., 2014; Lee and Jung, 2015; Jin et al., 2017).

Avoidance theory posits that people naturally seek pleasure and avoid pain (Elliot and Thrash, 2002). When information may cause unpleasant emotions, people choose to actively avoid that information (Sweeny et al., 2010), and consumers’ negative emotions toward a brand can be directly translated into actions against the brand, such as spreading negative word of mouth, avoidance, and retaliation (Khatoon and Rehman, 2021). Rebellious or resistant emotions can cause avoidance of advertising (Wan et al., 2015).

Researcher considered emotions a mediating variable between stimuli and their behavioral responses, with the final response being approach or avoidance (Mehrabian and Russell, 1974). Therefore, the mediating role of emotions has been widely used to study in the psychological mechanisms of consumer behavior. Positive perceptions can awaken positive emotions and thus promote purchase intentions (Wang, 2020). Negative perceptions can produce negative emotions and cause consumer avoidance behavior (Li, 2021). Petty argues that advertising influences consumer attitudes and behaviors toward advertising by affecting their emotions (Petty et al., 1988). Holbrook and Batra concluded that unreliable advertising content and time spent can influence consumers’ direct attitudes toward advertising through emotions (Olney et al., 1991). Therefore, the study proposes the following hypotheses:

H2: Negative emotions mediate the relationship between perceived risk and advertising avoidance.

H2a: Negative emotions mediate the relationship between perceived performance risk and advertising avoidance.

H2b: Negative emotions mediate the relationship between perceived privacy risk and advertising avoidance.

H2c: Negative emotions mediate the relationship between perceived time-loss risk and advertising avoidance.

H2d: Negative emotions mediate the relationship between perceived freedom risk and advertising avoidance.

#### The Moderating Role of Perceived COVID-19 Risk

Perceived COVID-19 risk is the individual’s subjective judgment and assessment of the severity, their susceptibility to infection, and controllability of the outbreak (Qian, 2015). From a psychological perspective, Some researchers found that the cognitive state of an individual to the exposure of an external stimuli, when inter-playing with the affective state to strengthen or weaken it, is possibly related to a consumer’s online behavior (Akram et al., 2017a). Thus when perceived COVID-19 risk is higher, consumers develop a health-first purchase motivation (Accenture China Consumer Insights Report, 2021). To reduce this risk people choose to reduce non-essential out-of-home actions (Wang et al., 2021a), reducing consumers’ offline access to information sources, and increasing the demand for information with high personal fitness (Fang, 2020; Accenture China Consumer Insights Report, 2021). Stimulated by a stressor, an individual will break the emotions generated by the original cognition, mobilize protective factors, and establish new beliefs or cognitions to reshape this equilibrium (Richardson, 2002). Approach-avoidance Theory (Roth and Cohen, 1986) suggests that the use of approach and avoidance strategies are not mutually exclusive. Stimulated by a situation, approach and avoidance may undergo a rapid transformation from avoidance of stress to coping with it. When perceived COVID-19 risk is high, consumers’ concerns may shift from negative emotions of online targeted advertising to perceived the risk of COVID-19, so their strategy toward online targeted advertising switches from avoidance to approach. Consumers may choose to shop online because of the perceived risk of COVID-19, and therefore enhance the demand for online advertising (Fang, 2020; Accenture China Consumer Insights Report, 2021). The stronger the consumers’ perceived COVID-19 risk, the weaker the effect of negative sentiment on online targeted advertising. Therefore, the study proposes the following hypothesis:

H3: Perceived COVID-19 risk moderates the relationship between negative emotions and advertising avoidance.

## Methodology

### Study Design

The methodology is based on a post-positivist research paradigm and used a cross-sectional quantitative questionnaire-based survey design. A third-party organization was used to recruit respondents from users who have been exposed to online targeted advertising. The questionnaire was self-administered, and completed voluntarily and anonymously. The questionnaire helped respondents understand and objectively answer whether they have been exposed to, or avoided targeted advertising, by providing specific explanations of precision advertising and advertising avoidance.

### Instrument Development

The measurement items for all variables were derived from well-established scales shown to have high reliability and validity. There were 7 latent variables and 33 measurement items (Table 2). Responses used a 5-point Likert scale, anchored by strongly disagree or strongly agree.

**TABLE 2:** Measurement items and constructs.

Construct	ID	Measurement items	Modified from source
Perceived performance risk	PER1	I think the product recommended by online targeted advertising is not reliable and may be false information	Lu, 2018; Liu, 2019; Qin, 2020
	PER2	You feel that the online targeted advertising recommendations do not match your expectations	
	PER3	I think online precision targeted advertising is worthless	
	PER4	I am concerned that clicking on online targeted advertising may threaten the security of your property	
Perceived privacy risk	PRR1	I think your privacy is being stealing for online targeted advertising	Dinev and Hart, 2006
	PRR2	I think that online targeted advertising interactions (such as likes, comments, or forwarding) will reveal your privacy	
	PRR3	I feel that the private data you have been obtained for delivering online targeted advertising could be misused. You feel that private data that is obtained by targeted advertising could be misused	
	PRR4	Privacy data that I feel has been obtained for delivering online targeted advertising could be made available to unknown individuals or companies without your knowledge or consent	
Perceived time-loss risk	TIR1	I think it will take a lot of time to browse online targeted advertising	Liu and Fu, 2014; Chen, 2020; Qin, 2020; Sun, 2020
	TIR2	I think it takes a lot of time to select and compare online targeted advertising	
	TIR3	I think it will take more time to return or replace goods due to online targeted advertising that do not match your expectations	
	TIR4	I feel that the time and place of online targeted advertising interrupts your personal time	
Perceived freedom risk	FRR1	I feel that online targeted advertising limits your freedom of choice	Dillard and Shen, 2005
	FRR2	I think online targeted advertising are trying to make decisions for you	
	FRR3	I feel online targeted advertising is trying to interfere with your choices	
	FRR4	I feel pressured by online targeted advertising	
Negative emotion	NEE1	I feel anxious about online targeted advertising	Quan and Ren, 2010
	NEE2	I find hate online targeted advertising	
	NEE3	I feel that online targeted advertising makes you angry	
Advertising avoidance	ADA1	I consciously ignored online targeted advertising	Cho and Cheon, 2004; Lei, 2019; Chao et al., 2020; Yang and Wen, 2020
	ADA2	I don’t pay attention to online targeted advertising based on my web activity	
	ADA3	I want to avoid technology tracking online behavior data	
;	ADA4	I wish advertisers would remove me from their targeted listings	
	ADA5	I would do something to avoid online targeted advertising	
Perceived COVID-19 risk	COV1	I am afraid of contracting the COVID-19	Chua et al., 2021; Venema and Pfattheicher, 2021
	COV2	I have a feeling that COVID-19 pandemic will break out all around me	
	COV3	I think it very frightening to be infected with the COVID-19	
	COV4	I feel that the current situation of the epidemic is worrying you	

The questionnaire was in three parts. The first part, the preface, introduced the researcher, stated the purpose of the survey, and defined the terms used. The second part elicited respondent demographic information. The third part contained the survey items, and used images and language to construct commonly encountered scenarios of targeted advertising.

### Data Collection

This online survey was carried out by a market research company in China with 2.6 million registered respondents. A total of 436 usable datasets were obtained. Table 3 shows the demographic characteristics of respondents included in the analysis. Of the respondents 37.61% are Bachelor degree holders, and 52.75% are postgraduate degree holders, and overall, respondents had a high education level.

**TABLE 3:** Sample descriptive statistics.

Variable name	Characteristics	Frequency	Proportion
Gender	Male	238	54.59%
	Female	198	45.41%
Age group	18–25	270	61.92%
	26–35	126	28.90%
	35–50	22	5.05%
	50 and above	18	4.13%
Education	High school (including junior college, technical school)	24	5.51%
	College and pre-college	18	4.13%
	Undergraduate	164	37.61%
	Graduate students	221	50.69%
	PhD and above	9	2.06%
Monthly income	Under 5,000 RMB	283	64.91
	5,000–8,000 RMB	53	12.16%
	8,000–10,000 RMB	29	6.65%
	10,000 RMB and above	71	16.28%
Career	Freelancer	27	6.19%
	Corporate staff	144	33.04%
	Students	257	58.94%
	Retirees	8	1.83%

## Data Analysis and Results

The data obtained from the questionnaire were analyzed using SPSS and AMOS. Covariance-based structural equation modeling (SEM) was used to test the research hypotheses.

### Analysis of the Measurement Model

#### Construct Validity and Reliability

Since the variables in this study are latent variables, a measurement model that correlates all latent variables was constructed to assess the quality of the data (Hair et al., 2017). Confirmatory Factor Analysis (CFA) was used to assess the reliability and validity of the latent variables in the model (Dimitrov, 2014). Factor loadings greater than 0.5 for each variable were considered acceptable; loadings greater than 0.7 were considered excellent. To confirm construct validity, the average variance extracted (AVE) score should be greater than 0.5. The Fornell-Larcker criterion was used to assess discriminant validity by determining if the arithmetic square root of the average variance extracted (AVE) was greater than the correlation between the latent variables (Hair et al., 2017). Table 4 shows the items used for each construct, Cronbach’s alpha values, composite reliability (CR) and AVE scores.

**TABLE 4:** Reliability and validity results of measurement mode.

Construct	Item	Factor loadings	Cronbach’s alpha	CR	AVE
Perceived performance risk	PER1	0.782	0.791	0.797	0.50
	PER2	0.686	0.930	0.931	0.771
	PER3	0.666	0.810	0.811	0.591
	PER4	0.677	0.896	0.897	0.687
Perceived privacy risk	PRR1	0.838	0.849	0.851	0.658
	PRR2	0.860	0.909	0.910	0.669
	PRR3	0.922	0.791	0.797	0.50
	PRR4	0.890	0.930	0.931	0.771
Perceived time-loss risk	TIR1	0.790	0.810	0.811	0.591
	TIR2	0.681	0.896	0.897	0.687
	TIR3	0.827	0.849	0.851	0.658
	TIR4	0.599	0.909	0.910	0.669
Perceived freedom risk	FRR1	0.842	0.791	0.797	0.50
	FRR2	0.828	0.930	0.931	0.771
	FRR3	0.863	0.810	0.811	0.591
	FRR4	0.779	0.896	0.897	0.687
Negative emotion	NEE1	0.837	0.849	0.851	0.658
	NEE2	0.871	0.909	0.910	0.669
	NEE3	0.717	0.791	0.797	0.50
Advertising avoidance	ADA1	0.810	0.930	0.931	0.771
	ADA2	0.769	0.810	0.811	0.591
	ADA3	0.857	0.896	0.897	0.687
	ADA4	0.860	0.849	0.851	0.658
	ADA5	0.789	0.909	0.910	0.669

The minimum value of Factor Loadings for the items used for each construct is 0.599, which is greater than 0.5 and significant; the minimum value of Cronbach’s alpha is 0.791; the Composite Reliability (CR) scores had a minimum value of 0.797, and AVE scores had a minimum value of 0.5, indicating that the variables had good reliability and validity.

From the above table (Table 5), it can be seen that the Square root of average variance extracted (AVE) of the six latent variables is greater than the correlations among latent variables, indicating that the data do not differ in terms of validity issues and therefore the data collected in this study are suitable for further analysis.

**TABLE 5:** Discriminant validity of measurement model.

Variable	PER	PRR	TIR	FRR	NEE	ADA
PER	0.500					
PRR	0.589***	0.771				
TIR	0.338***	0.424***	0.591			
FRR	0.649***	0.597***	0.285***	0.687		
NEE	0.559***	0.475***	0.356***	0.667***	0.658	
ADA	0.641***	0.729***	0.443***	0.635***	0.620***	0.669
$\sqrt{\text{AVE}}$	0.71	0.88	0.77	0.83	0.81	0.82

***p < 0.001, average variance extracted (AVE) is shown on the diagonal of the matrix.

#### Model Fit

The five indicators commonly used to evaluate model fit are chi-square (CMIN/DF), Goodness-of-Fit Index (GFI), Comparative Fit Index (CFI), Normed Fit Index (Byrne, 1998) and Root Mean Square Error of Approximation (RMSEA). The criteria are as follows: CMIN/DF ≤ 5 is reasonable; ≤3 is excellent. CFI, GFI, NFI ≥ 0.8 is reasonable; ≥0.9 is excellent; RMSEA ≤ 1 is reasonable; ≤0.6 is excellent (Marsh and Hocevar, 1985; Byrne, 1998; MacKinnon, 2008; Hair et al., 2010).

The model has good model fit with the following indices: CMIN/DF = 2.784, GFI = 0.884, NFI = 0.908, CFI = 0.939, and RMSEA = 0.064. Thus the data are ready for the next step of structural equation modeling.

### Structural Model and Hypotheses Testing

#### Model Fit

Before hypothesis testing, the fit of the structural equation model was evaluated. The criteria are the same as those used to evaluate the fit of the measurement model. In the structural equation, the independent variables are perceived performance risk, perceived privacy risk, perceived time-loss risk, and perceived freedom risk; the dependent variable is advertising avoidance, and the mediating variable is negative emotion. The fit indices of the structural model were: CMIN/DF = 4.434; GFI = 0.822; NFI = 0.85, CFI = 0.879, and RMSEA = 0.089; thus the overall model fit of the structural model is acceptable.

#### Hypothesis Testing

##### Correlation Analysis

AMOS software was used to evaluate the pathway system for testing of hypotheses 1 through 3, and the result are shown in Table 6. The Standard Coefficient, Standard Error (S.E.), Critical Ratios (C.R.) and *P*-values (P) were analyzed to determine if an explanation is supported or rejected (Filho et al., 2013). The results are shown in Table 6.

**TABLE 6:** Hypothesis testing (*n* = 436).

Hypothesis	Path	Coefficient	S.E	C.R.	*P*-value	Inference
H1a	PER→ADA	0.203	0.056	4.203	***	Supported
H1b	PRR→ADA	0.561	0.036	11.23	***	Supported
H1c	TIR→ADA	0.134	0.043	2.902	0.004**	Supported
H1d	FRR→ADA	0.165	0.043	2.967	0.003**	Supported

**p < 0.01, ***p < 0.001.

*PER, perceived performance risk; PRR, perceived privacy risk; TIR, perceived time-loss risk; FRR, perceived freedom risk; NEE, negative emotions; and ADA, advertising avoidance.*

Table 6 shows that Perceived Performance Risk (β = 0.203, *p* = 0.000), Perceived Privacy Risk (β = 0.561, *p* = 0.000), Perceived Time-Loss Risk (β = 0.134, *p* = 0.004), and Perceived Freedom Risk (β = 0.165, *p* = 0.003) have a positive effect on advertising avoidance. Thus, Hypotheses H1a, H1b, H1c, and H1d are supported.

##### Mediation Analysis

Mediation analysis explores influencing mechanisms or processes from independent to dependent variables (Cohen et al., 2014). This study used the bootstrapping method in AMOS, a popular method of testing the indirect effect (Shrout and Bolger, 2002). The mediating role of negative emotion in the relationship between perceived risk and advertising avoidance (H2) was determined by whether the 95% confidence interval of the mediating effect contains 0. If the 95% confidence intervals do not include 0 or two-tailed significance levels is significant, indicating mediating effect reaches a statistically significant level. The result of mediation for this research as shown in Table 7.

**TABLE 7:** Mediation testing results.

Hypotheses	Path	Bias-corrected Percentile 95% CI			Result
		Lower	Upper	*P*-value	
H2a	PER→NEE→ADA		Total effect		Supported
		0.126	0.393	0.001	
			Indirect effect		
		0.011	0.129	0.004	
			Direct effect		
		0.078	0.337	0.001	
H2b	PRR→NEE→ADA		Total effect		Rejected
		0.456	0.699	0.001	
			Indirect effect		
		−0.009	0.084	0.175	
			Direct effect		
		0.426	0.678	0.001	
H2c	TIR→NEE→ADA		Total effect		Supported
		0.061	0.284	0.004	
			Indirect effect		
		0.008	0.086	0.015	
			Direct effect		
		0.016	0.245	0.023	
H2d	FRR→NEE→ADA		Total effect		Supported
		0.188	0.477	0.001	
			Indirect effect	
		0.071	0.267	0.001	
			Direct effect		
		−0.002	0.365	0.054	

*n = 436; PER, perceived performance risk; PRR, perceived privacy risk; TIR, perceived time-loss risk; FRR, perceived freedom risk; NEE, negative emotion; ADA, advertising avoidance.*

AMOS was used to produce 2000 bootstrap samples with 95% BC Confidence Level. Perceived Performance Risk affects advertising avoidance through the mediating effect of negative emotions, 95% confidence interval of indirect effect [0.011, 0.129] does not contain 0, *p*-value = 0.004 < 0.05, indirect effects reach statistical significant level. H2a was supported. The 95% confidence interval of total effect [0.126, 0.393] did not contain 0, *P*-value = 0.001 < 0.05, and the 95% confidence interval of direct effect [0.078, 0.337] did not contain 0, *P*-value = 0.001 < 0.05, therefore total effect and direct effect were both present. Negative emotions partially mediate the relationship between perceived performance risk and advertising avoidance.

Perceived privacy risk influenced advertising avoidance through a mediating effect of negative emotions. The 95% confidence interval for the indirect effect [−0.009, 0.084] contained 0, *p*-value = 0.175 > 0.05, and the indirect effects was not significant level. H2b was not supported. The 95% confidence interval for total effect [0.456, 0.699] did not contain 0, *p*-value = 0.001 < 0.05. The 95% confidence interval for direct effects [0.426, 0.678] did not contain 0, *p*-value = 0.001 < 0. 05, total and direct effects were present, therefore, negative emotions did not mediate the relationship between perceived privacy risk and advertising avoidance.

The perceived risk of time-loss influenced advertising avoidance through a mediating effect of negative emotions, the 95% confidence interval for the indirect effect [0.008, 0.086] did not contain 0, *p*-value = 0.015 < 0.05, and the indirect effects reached a statistically significant level. [0.061, 0.248] did not contain 0, *p*-value = 0.004 < 0.05, 95% confidence interval for direct effect [0.016, 0.245] did not contain 0, *p*-value = 0.023 < 0. 05; therefore both total and direct effects were present and negative emotions partially mediated the relationship between perceived risk of time-loss and advertising avoidance.

Perceived freedom risk influenced advertising avoidance through a mediating effect of negative emotions, the 95% confidence interval for the indirect effect [0.071, 0.267] did not contain 0, *p*-value = 0.001 < 0.05, and the indirect effect reached statistical significance [0.188, 0.477] did not contain 0, *p*-value = 0.001 < 0.05, and the 95% confidence interval for the direct effect [−0.002, 0.365] did not contain 0, *p*-value = 0.054 close to 0.05, so the total effect was marginally significant. Thus, negative emotions fully mediated the relationship between perceived freedom risk and advertising avoidance.

##### Moderation Analysis

Moderation analysis studies how the effect of one variable on another variable changes under different conditions, i.e., different levels of the moderating variable. We used Multiple-Group Analysis in AMOS to verify whether the effect of negative emotion on advertising avoidance is the same when perceived COVID-19 risk is high or low, to determine if this perceived risk moderates the relationship between negative emotion and advertising avoidance. The 27% highest on the criterion measure of perceived COVID-19 risk are classified as the high-risk perceived COVID-19 group, and the 27% lower are classified as the low-risk perceived COVID-19 group (Kelley, 1939). High-risk perceived COVID-19 group and low-risk perceived COVID-19 group were set in AMOS. By model comparison between unlimited model and constraint model with High-risk perceived COVID-19 = low-risk perceived COVID-19 parameter constraints, *p*-value was 0.008, indicating that there was significant differences between the two models. Thus, perceiving COVID-19 risk moderated the relationship between negative emotions and advertising avoidance. H3 was supported.

## Conclusion

### Discussion

This study provides insights into consumer perceptions of the risks of online targeted advertising, whether these perceived risks influence advertising avoidance; and identified the mechanism of influences of perceived risks upon advertising avoidance. The keys findings are as follows:

We first discuss the important effects of perceived risk of online targeted advertising on advertising avoidance. Perceived performance, privacy, time-loss and freedom risks positively influence online targeted advertising avoidance, in decreasing order of perceived privacy risk (β = 0.561), perceived performance risk (β = 0.203), perceived freedom risk (β = 0.165), and perceived time-loss risk (β = 0.134). Overall, perceived privacy risk is the most significant predictor of advertising avoidance. Internet companies use advanced internet and computing technologies to collect user data for increasingly accurate personalized recommendations (Kim and Kim, 2017). There is a paradox for users, who may enjoy the convenience of accurate recommendations (Shareef et al., 2017), whilst also feeling threatened by collection of user information (Chen et al., 2019; Labrecque et al., 2021), restriction of freedom of choice, etc (Aguirre et al., 2015; Newell and Marabelli, 2015). Users develop advertising avoidance when they perceive the threat of online targeted advertising. This findings are line in technology threat avoidance theory (TTAT), approach-avoidance theory, psychology reactance theory, and are consistent with literature that shows user risk perception affects their behavior and motivation (Miyazaki and Fernandez, 2001; Aguirre et al., 2015; Newell and Marabelli, 2015; Xiao and Benbasat, 2018; Chen et al., 2019; Kurtz et al., 2021; Labrecque et al., 2021). The path coefficient of the effect of user perceived privacy risk on avoidance behaviors is much larger than the other three perceived risks, indicating that perceived privacy risk has a predominant effect on online targeted advertising avoidance. This finding is consistent with those of Wang et al. (2021b) that consumer privacy concerns are the biggest impediment of online targeted advertising today (Jiang and Wang, 2020).

Secondly, mediation analysis found that negative emotions partially mediate the relationship between perceived performance, time-loss and freedom risks and online targeted advertising avoidance. In other words, in the mediation model with negative sentiment as a mediating variable, perceived risks directly influence avoidance and also cause negative sentiments, resulting in avoidance. This finding is consistent with the literature, where perceptions of risk can generate a range of negative emotions that influence consumer behavior and attitudes (Mehrabian and Russell, 1974; Petty et al., 1988; Olney et al., 1991; Li, 2021). Of particular note is that contrary to expectations, negative emotions have no mediating effect between perceived privacy risk and online targeted advertising avoidance. A plausible explanation is that close to 90% of the study respondents study had a Bachelor degree or higher. According to Coping Behavior Theory (CBT), coping styles that consumers may adopt when they experience negative emotions triggered by privacy concerns are divided into two categories, but the specific situations in which consumers will choose which coping style depends on the resources available to them (Zhang and Li, 2018). Educated people may be more familiar with the laws and regulations on personal data protection, such as the Data Security Law of the People’s Republic of China and the Personal Information Protection Law, or understand that they can deny merchants access to personal information through web browser settings. If consumers have sufficient resources (or higher controllability) to deal with negative events directly, they will actively seek solutions, thus ameliorating negative outcomes. There are also ways to mitigate negative emotions (Gross, 1998), thus when perceiving the privacy risks of online advertising, more highly educated consumers seek solutions and mitigate negative emotions. Another possible explanation is associated with the large proportion of young people in the study respondents, in that young consumer tend to have an optimistic attitude toward privacy (Zhang et al., 2020).

Thirdly, moderation analysis found that perceived COVID-19 risk moderated the relationship between negative emotions and online targeted advertising avoidance. Consumers’ perceptions of the continued health threat of COVID-19 is likely to enhance their defense motivation, and reduce their travel while increasing their demand for online information. The motivation for danger control enhances the information-seeking process (Witte, 2009). This inference is strongly supported by China’s economic statistics, that show that annual retail sales of consumer goods declined 3.9% in 2020 compared to 2019, whereas online sales grew by 10.9% over 2019 (China Government Network, 2020), and that internet advertising revenue for 2020 grew by 13.85% over 2019 (China Economic, 2021). COVID-19 associated stressors are likely to have spurred consumers to overcome negative sentiments toward online targeted advertising and shifted them from avoidance to acceptance.

### Implications for Theory

The contributions of this study to theory are five-fold. Firstly, this study combined brand avoidance theory, approach-avoidance theory and SOR theory to construct a new theoretical framework for advertising avoidance. This provides a theoretical reference for future research on online targeted advertising.

Secondly, the study extends brand avoidance theory (Knittel et al., 2016) that posits that when consumers have negative emotions about advertisements, they avoid them. However, the theory does not explain why consumers develop negative emotions. By using consumers’ negative perceptions as the antecedent variable for negative emotions to explain why consumers develop negative emotions that lead to advertising avoidance, this study complements brand avoidance theory.

Thirdly, extending the SOR model. This study extends the SOR theory by adding perceived COVID-19 risk as a moderating variable to explain whether consumer perception of COVID-19 risk moderates the effect of negative emotions on targeted advertising avoidance in the post-pandemic era.

Fourthly, the applicability of perceived risk theory is expanded. The literature review found that perceived risk theory is mainly used to study consumers’ online shopping behavior. This study combines perceived risk theory with brand avoidance theory for the study of consumer advertising avoidance, which expands the application scope of perceived risk theory.

Fifthly, extending the literature by proposing an additional dimension of perceived risk, which has financial, functional, physical, psychological, social, and time-loss dimensions. Because these dimensions can vary depending on the industry or product studied (Peter and Ryan, 1976; Liu et al., 2015; Yang et al., 2020), this study focused on consumer advertising avoidance; therefore, combining the research context and psychology reactance theory, this study argues that online targeted advertising interferes with consumers’ goals and has the potential to limit their action choices, which is important for making consumers perceive freedom risk (Edwards et al., 2002). Thus the theory of perceived risk may be extended by adding perceived freedom risk as an additional dimension.

### Implications for Managers

This study analyzes the mechanism of influence on online targeted advertising behavior from the perspective of consumers’ perceived risk. It has some reference value for governments, platforms and advertisers.

For the government: The results of the study show that consumers’ perception of privacy risk is the biggest influencing factor on advertising avoidance, and perceived freedom risk also significantly affects advertising avoidance. Every user gets remunerations when the relationships are bound by respect, quality and fulfillment (Umair et al., 2018). Therefore, the government needs to strengthen legislation so that consumers’ personal information and private data are actually respected and protected in a reasonable manner. At the same time, it should further strengthen the protection of consumers’ freedom rights, and promote the appropriate handling and use of personal information resources within the framework of the legal principle of “legality, legitimacy and necessity,” while ensuring that citizens’ personality and privacy rights are not infringed upon.

For advertisers: First of all, advertisers need to improve the advertisement content in terms of mitigating the performance risk and time-loss risk perceived by consumers. The content of advertisements should be as comprehensive and precise as possible to provide information about the goods like online targeted advertising with precised individual’s propensity shopping vouchers as it drives the purchase (Akram et al., 2020), and avoid exaggerating the quality and functions of the goods or services, causing consumers to think that they are not in line with expectations. It should also be noted that the content length and video duration should not be too long and should be focused so that consumers in need can have an intuitive understanding of the promoted products and services in a short time and reduce the time cost of browsing targeted advertisements. Secondly, advertisers should choose the right platform, considering the suitability of the scenario with their own products and the reliability of the platform, to further reduce consumers’ perceptions of performance and time-loss risks. Finally, during the COVID-19 pandemic, the impact of sudden public emergencies on consumers and the market is continuous. Advertisers can help themselves resume normal operations through online targeted advertising.

For platform enterprise: Platforms enterprise need to improve their advertising platforms in terms of mitigating the perceived privacy and freedom risks to consumers. It is important to collect and use consumer-generated data under the scope of legal compliance, and to find a balance between the degree of accuracy and protection of personal privacy. Reinforce consumer information usage instructions and privacy settings. Add settings related to the “right to opt out” and “right to delete,” so that consumers can see their tagged tags, and they can also freely choose to opt out or delete these tags to improve consumer recognition of the platform’s security. At the same time, the platform cannot repeatedly push homogeneous advertising content in pursuit of economic benefits, thus alleviating the perceived risk of consumer freedom and time-loss.

In conclusion, this study is an important guide for enterprises or platforms to develop advertising strategies that mitigate consumers’ risk perceptions and negative emotions and improve the effectiveness of targeted advertising. It also provides new ideas for enterprises to find a balance between the benefits and risks of precision advertising.

### Limitations and Future Research

#### Limitations

Although this study contributes to the research on online targeted advertising, there are shortcomings.

Firstly, as study respondents were from China, recruited through “Wen Juan Xing” in China, the findings may not be applicable to other cultures. The findings should also be interpreted in cause the respondents were generally more highly educated than the general population.

Secondly, this study used a quantitative research approach, and all variables were selected with reference to previous literature, and the items of the questionnaire were adapted to mature domestic and international scales, so there may be incomplete coverage of the constructs and items.

Thirdly, online targeted advertising is delivered via different online platforms, including social, e-commerce, short video, and search platforms. The platform type may influence consumers’ avoidance behavior of advertising, studies may need to identify this influence and compare platform types.

Fourthly, this study limits itself to perceived COVID-19 risk as a restricted moderator to moderate the relationship between negative emotions and advertising avoidance. Thus, the study excludes some of the potential factors such as personal factor and environment factor that may be moderate the influence of negative emotions and advertising avoidance.

#### Further Research

The research can be extended in at least three areas: (1) To aid instrument development qualitative interviews of consumers of advertising should provide insights into mechanisms of advertising avoidance in different cultural contexts. Sample sizes can be increased and can compare different populations, such as those of developed and developing countries. (2) Qualitative research approach, such face to face interviews, can be adopted. (3) Avoidance of advertising on different online media platforms can be compare. (4) Future studies can add other possible mediating and moderating variables into the research framework.

## Data Availability Statement

The raw data supporting the conclusions of this article will be made available by the authors, without undue reservation.

## Author Contributions

YQJ: data curation and software. HJW: formal analysis and project administration. XLY: investigation and writing—original draft. HJW and XLY: methodology. ARA, GQT, and JYD: supervision. ARA and GQT: validation. JYD and YQJ: writing—review and editing. All authors read and agreed to the published version of the manuscript.

## Conflict of Interest

The authors declare that the research was conducted in the absence of any commercial or financial relationships that could be construed as a potential conflict of interest.

## Publisher’s Note

All claims expressed in this article are solely those of the authors and do not necessarily represent those of their affiliated organizations, or those of the publisher, the editors and the reviewers. Any product that may be evaluated in this article, or claim that may be made by its manufacturer, is not guaranteed or endorsed by the publisher.
